# Effects of the Energy Density on Pores, Hardness, Surface Roughness, and Tensile Characteristics of Deposited ASTM 316L Specimens with Powder-Bed Fusion Process

**DOI:** 10.3390/ma15196672

**Published:** 2022-09-26

**Authors:** Ho-Jin Lee

**Affiliations:** Smart Manufacturing Technology R&D Group, Korea Institute of Industrial Technology, Daegu 42994, Korea; hlee3@kitech.re.kr; Tel.: +82-53-580-0147

**Keywords:** powder bed fusion, ASTM 316L, mechanical properties, energy density, additive manufacturing

## Abstract

Powder bed fusion (PBF) is a typical metal-AM process. Studies on the process parameters are required to fabricate the desired shape without defects in the PBF process. The aim of this study is to investigate the effects of energy density on the pore, hardness, surface roughness, and tensile characteristics of deposited ASTM 316L specimens using a powder-bed fusion process. Twenty-seven types of specimens with different laser powers, scanning speeds, and overlap ratios were fabricated using the PBF process. The effects of the energy density on the porosity, hardness, surface roughness, tensile strength, and fracture properties of ASTM 316L specimens were examined. The relationships between these properties and energy density are discussed. A critical energy density level was suggested as 79 J/mm^3^ considering these characteristics. With the critical energy density level, relative density, surface roughness (Ra) and hardness were observed 99.5%, 1.2 μm, and 240 HV, respectively. Additionally, these characteristics were improved with increasing energy density. Five representative conditions were chosen to fabricate tensile specimens with the ASTM 316L powder through the PBF process. Tensile characteristics, including ultimate strength, yield strength, strain, and fracture shape, were examined for different energy densities. The best tensile characteristics were observed with the highest energy density level of 155 J/mm^3^.

## 1. Introduction

Additive manufacturing (AM) is the layer-by-layer deposition of materials, which is different from the material removal and forming processes. The AM processes, such as 3D printing and rapid prototyping, have various advantages including a low buy-to-fly ratio, accessibility of technology, possibility of fusion with different technologies, manufacturability of complex geometrical 3D shapes (design freedom), adaptability of various materials for a single product, and manufacturability of functional gradient products [1,2,3]. AM technology is used in various fields worldwide, such as education, aerospace, architecture, mechanical engineering, art, and housewares. The range of tasks for AM technology is steadily expanding with the development of AM technology [1,2,3]. Powder bed fusion (PBF) is a typical metal AM process. The PBF process uses spherical metallic powder material [1,4,5,6]. The desired solid part is fabricated by repeating several sequences, such as powder feeding, melting of the fed powder layer, and solidification of the melted powder [5,6,7]. During the deposition process, a high-density energy source is used to melt powder materials, such as laser and electron beams. The PBF process can be classified into selective laser melting (SLM), selective laser sintering (SLS), direct metal laser sintering (DMLS), electron beam melting (EBM), and electron beam sintering (EBS), depending on the type of energy source and material fusion characteristics [1,2,3,4,5,6,7,8]. The PBF process has various process parameters, including laser power, scanning speed, layer thickness, beam diameter, hatching distance, hatching angle, ambient temperature, and temperature of the building plate [9,10,11,12]. The major defects in the PBF process are delamination of the layer, poor mechanical properties, pores, and deflection [5,8,10,11,12,13]. The delamination defect is the delamination between the layers owing to the lack of fusion or an inappropriate tool path. Poor mechanical properties are observed when the applied parameter conditions are unsuitable. Pores are also created with poor parameter conditions, such as a weak energy density, to melt the deposition material fully. Deflection defects occur with a poor design of the tool path and support. Such defects occur when a combination of the process parameters listed above is unsuitable [11,12,13,14,15]. Hence, studies on the process parameters are needed to fabricate the desired shape without defects in the PBF process. Many researchers are conducting studies to manufacture high-quality products using the PBF process.

Cai et al. studied the parameter optimisation of a titanium alloy using the PBF process [16]. The laser power, scanning speed, layer thickness, and hatching distance were chosen as the experimental parameters in their study. They discussed the effects of process parameters on the microstructure and mechanical properties of the deposited specimen. Lo et al. studied the optimised hatch distance in a double-scanning-track PBF process [17]. They obtained a single-track process map for different laser powers and scanning speeds. They discussed five types of representative state. The overlap ratio on a single bead was an important parameter in the fabrication of high-quality parts. The overlap ratio is calculated from the dimensions of a single bead.

Stainless steel is in high demand in various industries owing to its superior weldability and excellent thermal and corrosion resistance [18,19]. ASTM 316L is austenitic stainless steel with a low carbon rate (less than 0.03%) [19]. Due to the high industrial demand for ASTM 316L, studies on the production of ASTM 316L specimens and products using the PBF process are being actively conducted. The importance of the parametric setting of the PBF process for ASTM 316L has been reported previously [1,2,3,4,5,19,20,21,22].

Peng et al. conducted a study on the influence of energy density on the energy demand and porosity of 316L Stainless steel fabricated via selective laser melting [20]. Liverani et al. investigated the effects of the PBF process on the microstructure and mechanical properties of ASTM 316L [21]. They discussed the changes in the tensile and fault characteristics for different laser powers, hatch distances, and building directions. Larimian et al. reported the effect of energy density on the microstructure and mechanical properties of a specimen deposited by the PBF process [22]. They discussed the mechanical properties at different energy density levels. However, a small number of investigations have been carried out on the effect of the process parameters on the characteristics of the deposited ASTM 316L specimen with the PBF process.

In this study, we aimed to investigate the effects of energy density on the pore, hardness, surface roughness, and tensile characteristics of ASTM 316L specimens deposited using a powder-bed fusion process. Twenty-seven types of spacemen with different laser powers, scanning speeds, and overlap ratios were fabricated using the PBF process. The effects of process parameters, including laser power, scanning speed, and overlap ratio, on the fabrication characteristics of ASTM 316L specimens through the PBF process are discussed. These process parameters were simplified with the energy density for a systematic evaluation. The effects of the energy density on the porosity, hardness, surface roughness, tensile strength, and fracture properties of ASTM 316L specimens were examined. The relationships between these properties and energy density are discussed. Five representative conditions were chosen to fabricate tensile specimens with ASTM 316L powder through the PBF process. Tensile characteristics, including ultimate strength, yield strength, strain, and fracture shape, were examined for different energy densities. The critical energy density level is discussed considering these characteristics.

## 2. Experimental Set-Up and Method

**Figure 1:**
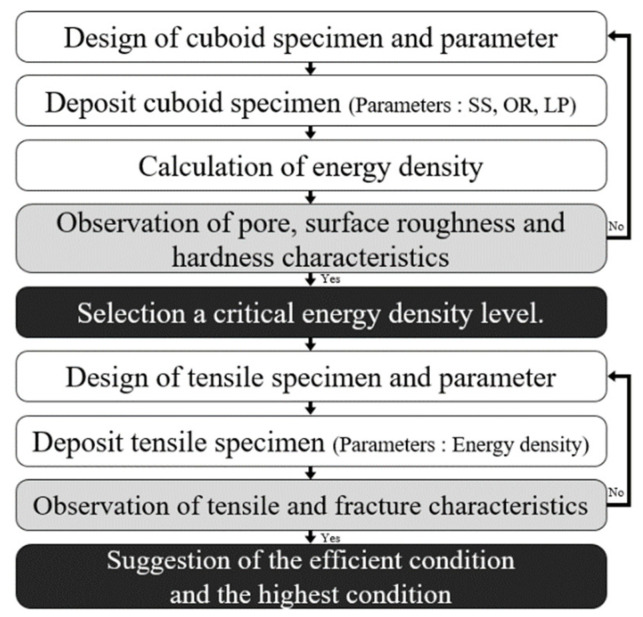
Flow chart of experiments.

**Figure 2:**
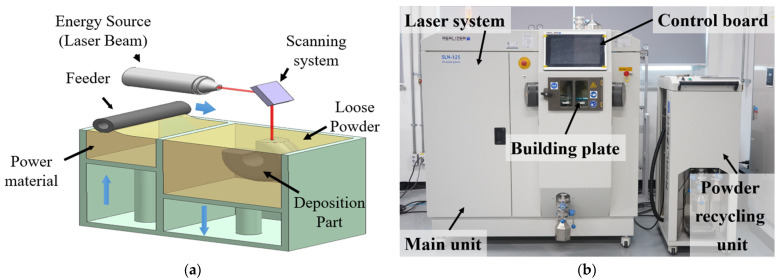
Conceptual diagram and experimental set-up of PBF process: (**a**) conceptual diagram of PBF process and (**b**) experimental set-up of PBF process.

The variations in the surface roughness, porosity, and hardness according to the energy densities were examined for the deposited cuboid specimens. The tensile specimens were produced under certain process conditions used to produce cuboid specimens. These tensile specimens were then used to examine the yield strength, ultimate strength, elongation, and failure properties based on the energy densities used in the additive process. Figure 1 shows a flow chart of experiments. Figure 2 shows a picture of the experimental equipment. Figure 2a shows a conceptual diagram of the PBF process used in the experiments. The PBF process forms the desired shapes by spreading loose powder over a building plate. The powder is then selectively melted locally using a heat source. The melted powder is then solidified. Laser and electron beams are generally used as heat sources in the PBF process. The powder over the building plate was transported from the power supply unit using a powder feeder. Figure 2b shows the SLM-125 by ReaLizer used in the experiments. The type, diameter, wavelength, and maximum power of the heat source used in this equipment are ytterbium fibre laser, 72 μm 1076 nm, and 400 W, respectively. The maximum dimensions of the building space are 125 mm × 125 mm × 200 mm. The building plates were fabricated using ASTM 1045 material with dimensions of 125 mm × 125 mm × 100 mm. A chamber system was used to control the oxidation and temperature during the process.

The process of producing the deposition specimens consisted of preheating, powder supply, deposition, cooling, and ejection. In the preheating stage, oxygen remaining in the chamber was lowered to at most 0.3% by injecting nitrogen gas into the chamber to maintain an inert gas atmosphere. The building plate was then heated at 150 °C for 30 min to improve the fusibility of the powder during the additive process. The temperature was maintained for 1 h after heating to induce a thermal equilibrium state in the system. In the powder-supplying stage, the powder supplied to the chamber is transported onto the preheated building plate by rotating the powder feeder. During the deposition process, the powder spread over the building plate was selectively melted using a laser system and then solidified. This process was repeated to obtain the desired shape. The cooling process was performed for 3 h in a gas atmosphere to stabilise the structure of the specimens after they were produced and to suppress high-temperature oxidation when ejecting the specimens.

**Figure 3:**
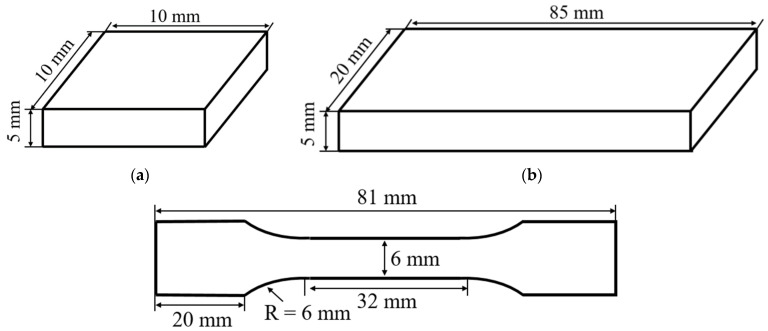
Design of specimens: (**a**) design of cuboid deposition specimen; (**b**) design of tensile specimen before cutting; and (**c**) design of tensile specimen after cutting.

The dimensions of the cuboid deposition specimens produced by the PBF process are 10 mm × 10 mm × 5 mm, as shown in Figure 3a. The dimensions of the tensile specimens produced by the PBF process are 85 mm × 20 mm × 5 mm, as shown in Figure 3b. The tensile specimens were machined into a dog bone shape with dimensions of 81 mm × 12 mm × 2 mm, as shown in Figure 3c.

The variables applied in the cuboid deposition experiments included laser power, scanning speed, and bead overlapping ratio. The laser power, scanning speed, and bead overlapping ratio ranges used in the cuboid deposition experiments were 100–200 W, 500–1500 mm/s, and 0–50%, respectively. Specimens for 27 conditions were produced using combinations of experimental variables. 27 conditions were calculated from three variables with three levels as full factorial. 

**Figure 4:**
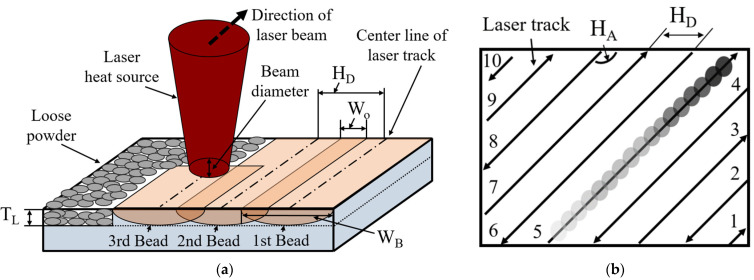
Conceptual diagram of a cuboid deposition experiment: (**a**) conceptual diagram of deposition process and (**b**) conceptual diagram of the toolpath.

Figure 4a shows a conceptual diagram of the cuboid deposition process. Figure 4b shows a conceptual diagram of the toolpath. An initial additive hatching angle (HA) of 67° was applied. After the first layer, the HA was repeatedly rotated by 67° to avoid deposition path overlap in the height direction [23].

To simplify a significant number of variables, energy density was used as a significant consideration. The general energy density formula for the heat input used in the deposition is given by Equation (1), where ED, LP, T_L_, SS, and H_D_ are the energy density, laser power, thickness between the layers, scanning speed, and hatching distance, respectively. A T_L_ of 30 μm was used [22].

(1) $$\mathrm{ED}\left(J/{\mathrm{mm}}^{3}\right)=\frac{\mathrm{LP}\left(W\right)}{T_{L}\left(\mathrm{mm}\right)\times \mathrm{SS}\left(\mathrm{mm}/s\right)\times H_{D}\left(\mathrm{mm}\right)}$$

The bead overlapping ratio was calculated using Equations (2) and (3), where W_O_ and W_B_ are the overlapping distance and bead width, respectively.

(2) $$\mathrm{OR}\left(\%\right)=\frac{W_{O}\left(\mathrm{mm}\right)}{H_{D}\left(\mathrm{mm}\right)}$$

(3) $$W_{O}\left(\mathrm{mm}\right)=W_{B}\left(\mathrm{mm}\right)-H_{D}\left(\mathrm{mm}\right)$$

As the bead width varies according to the laser power and scanning speed, the widths of the beads created in the laser power range of 100–200 W and scanning speed range of 500–1500 mm/s were measured experimentally to calculate the bead overlapping ratio. The calculation results of the energy density obtained by applying the 27 conditions used in the cuboid specimen fabrication are listed in Table 1. In this study, the energy density range used for the specimens was 25–155 J/mm^3^.

**Figure 5:**
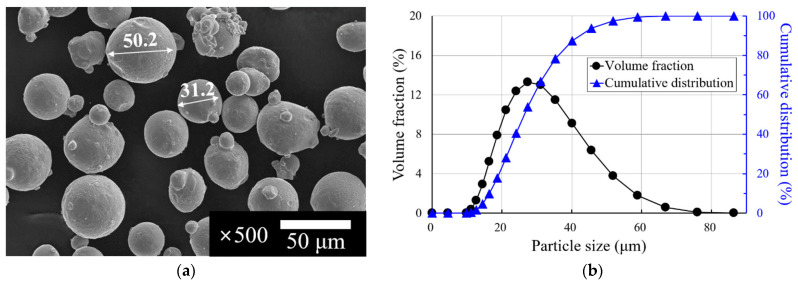
Shape and size distribution of ASTM 316L powder: (**a**) shape of ASTM 316L powder and (**b**) volume fraction and cumulative distribution of ASTM 316L powder.

The ASTM 316L (MetcoAdd 316L-A, Oerlikon metco, Wohlen, Switzerland) powder used in the deposition was spherical with an average particle size of 35 μm. Figure 5a shows a scanning electron microscopy (SEM) image of the powder used in the experiments. SEM images were obtained using a SU8020 field emission (FE)-SEM (Hitachi, Tokyo, Japan). Figure 5b shows the results of measuring the particle size distribution of the powder used in the experiments, which was measured using a particle size analyser, Mastersizer-3000 (Malvern Panalytical, Malvern, UK). The chemical composition of deposition and substrate material is shown in Table 2.

The surface roughness, porosity, and hardness of cuboid-deposited specimens were measured. The surface roughness was measured using a 3D laser microscope (LEXT OLS4100-SAA, Olympus, Tokyo, Japan) and evaluated using Ra and Rz. The Ra and Rz formulae are represented in Equations (4) and (5), where l, P, and V are the measurement length, maximum height from the centreline, and maximum depth from the centreline, respectively.

(4) $$R_{a}=\frac{1}{l}\int_{0}^{l}\left|f\left(x\right)\right|\mathrm{dx}$$

(5) $$R_{Z}=\frac{1}{5}\overset{5}{\underset{n=1}{\sum }}\mathrm{Pn}+\frac{1}{5}\overset{5}{\underset{n=1}{\sum }}\mathrm{Vn}$$

Pores were measured using a Stemi508 stereomicroscope (Zeiss, Oberkochen). The sectional porosity was calculated according to Equation (6), where AP and AI were areas of pore and inspection, respectively.

(6) $$\emptyset \left(\%\right)=A_{P}\left({\mathrm{mm}}^{2}\right)/A_{I}\left({\mathrm{mm}}^{2}\right)$$

The sectional porosity was measured while repeatedly polishing the surfaces. The average porosity was used for discussing the energy density effects. The number of measured sections and points was 3 and 30, respectively. The average pore size was also measured using a stereomicroscope. Average pore size was calculated from the sectional porosity images.

Hardness was measured using a hardness tester, HR-500 (Mitutoyo, Kawasaki, Japan). Sections of the deposited specimens were cut. Their hardness at nine points was measured to obtain the average hardness.

The yield strength, ultimate strength, elongation, and failure shape of the tensile specimens were measured. The yield strength, ultimate strength, and elongation were measured using a 400-KN 5988 universal testing system (Instron, Norwood, USA). Images of failure shapes were obtained using SEM.

## 3. Results and Discussion

### 3.1. Morphology of Specimen according to Process Parameters

**Figure 6:**
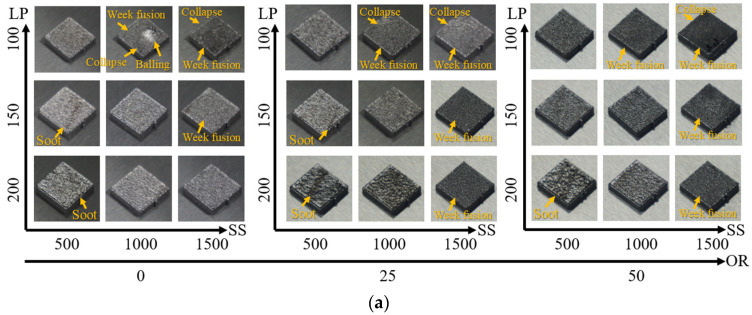

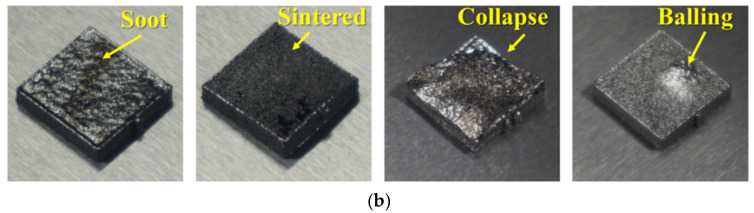
Deposition results according to process parameters: (**a**) morphology of deposited ASTM 316L specimen according to process parameters and (**b**) surface defect mode of deposited specimens.

Cuboid specimens for the 27 conditions according to the laser power, scanning speed, and bead overlapping ratio variations were deposited using the PBF process. Figure 6a shows trimetric images of the 27 specimens. Similar defect characteristics are observed compared with previous research works [7,8]. Surface soot, weak fusion, collapse, and balling defects were observed under certain deposition conditions, as shown in Figure 6b. The surface soot was characterised by soot stains on the specimen surfaces along the fusion path. Weak fusion resulted from incomplete melting and bonding in the sintered form. Shape collapses occurred when the specimens were produced in undesirable shapes by tilting or partial collapse. The balling defects had round shapes, such as balls created on some specimens.

Surface soot was found regardless of the bead overlapping ratio when the laser power was 200 W, and scanning speed was 500 mm/s. Surface soot was also found under identical conditions, potentially caused by the over-melted regions.

Collapses were found regardless of the bead overlapping ratio when the laser power was 100 W, and scanning speed was 1500 mm/s. However, these collapses were found only when the bead overlapping ratio was 0% and 25%. The laser power was 100 W, and scanning speed was 1000 mm/s.

Weak fusion defects were found regardless of the laser power and bead overlapping ratio when the scanning speed was 1500 mm/s. Weak fusion defects were also observed only when the laser power was 100 W at a scanning speed of 1000 mm/s. The weak fusion and collapse defects were caused by the insufficient heat input capacities used in the fusion.

Balling defects (some regions swelling like balls) were observed when the laser power was 100 W; the scanning speed was 1000 mm/s, and the bead overlapping ratio was 0%. Such defects appear owing to the repeated weak fusion and collapse defects.

### 3.2. Porosity Characteristics according to Energy Density

**Figure 7:**
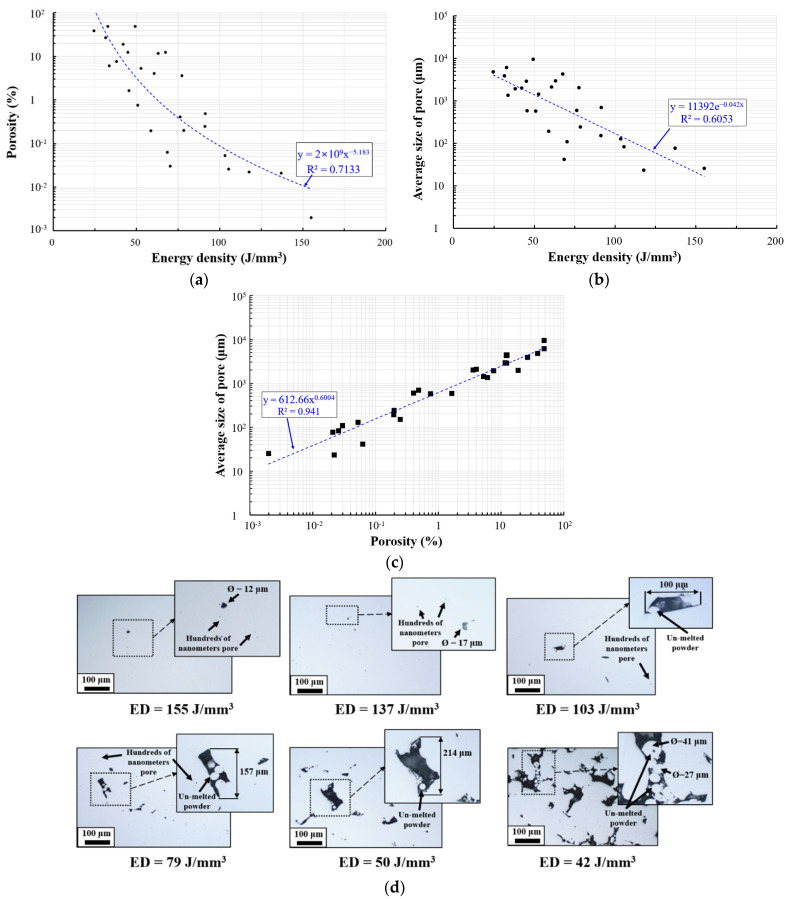
Sectional pore characteristics of deposited specimen for different energy density (J/mm^3^): (**a**) plot of sectional porosity (%) according to energy density (J/mm^3^); (**b**) plot of average size of pore (μm) according to energy density (J/mm^3^); (**c**) plot of average size of pore (μm) according to porosity (%); and (**d**) morphology of the internal pore according to the energy density (J/mm^3^).

The pore properties of the 27 specimens deposited by the PBF process were evaluated considering the energy density used in specimen deposition. Greco et al. presented that the maximum sectional porosity of AISI 316L specimen is above 5% when energy level is 119 J/mm^3^ [8]. However, the improved sectional porosity characteristics results were obtained in this work. Figure 7a shows the results of measuring the sectional porosity variation according to the energy density used in the specimen deposition. Sectional porosity of 0.1% or lower was observed when the energy density was 103 J/mm^3^ or higher. The lowest sectional porosity of 0.002% was observed when the energy density was 155 J/mm^3^. A sectional porosity of 0.2–0.5% was measured when the energy density was between 79 J/mm^3^ and 103 J/mm^3^. Unstable behaviour with a sectional porosity of 0.03–12% was observed when the energy density was between 50 J/mm^3^ and 79 J/mm^3^. When the energy density was lower than 50 J/mm^3^, the sectional porosity was 1.6–48%, corresponding to specimens similar to porous structures.

The sectional porosity variation according to the energy density in the test range can be expressed as a decreasing curve, represented by the equation Y = 2 × 10^9^x^−^^5.183^, with a confidence level of 71%. These findings indicate a significant correlation between the energy density used in specimen deposition and sectional porosity of the specimens. The higher the energy density, the lower the porosity. The energy densities must be at least 79 J/mm^3^ and 103 J/mm^3^ to produce ASTM 316L specimens with relative densities of 99.5% and 99.9%, respectively, using the PBF process.

Figure 7b shows the results of measuring the variation in the average pore size according to the energy density used in the specimen deposition. The average pore size was 130 μm or lesser at an energy density of 103 J/mm^3^ or higher. The lowest average pore size (25 μm) was observed when the energy density was 155 J/mm^3^. The average pore size was 150–688 μm when the energy density was between 79 J/mm^3^ and 103 J/mm^3^. Unstable behaviour with an average pore size between 41 and 4200 μm was observed when the energy density was between 50–79 J/mm^3^. The average pore size ranged from 580 μm to 9438 μm when the energy density was lower than 50 J/mm^3^. It was 16–270 times larger than the average particle size of the powder. The average pore size decreased with increasing energy density in the test range. The curve can be expressed using the exponential function Y = 11,392 × 10^−0.042x^, with a confidence level of 60%.

These results indicate a significant correlation between the energy density used in ASTM 316L specimen deposition and average pore size of the specimens. The higher the energy density, the smaller the average pore size of the specimens. The energy density must be at least 155 J/mm^3^ to produce high-quality ASTM 316L specimens with average pore sizes smaller than the average particle size of supplied powder. The energy density must be at least 103 J/mm^3^ to produce specimens with an average pore size four times smaller than the average powder particle size. When the energy density is 79 J/mm^3^, an excellent relative density of 99.5% is obtained. However, the pore size was 20 times the average particle size of the powder.

Figure 7c shows the correlation between the average pore size and sectional porosity from the measurement results of the sectional porosity and average pore size variation according to the energy density used in fabricating the specimens. The average pore size variation according to the sectional porosity can be expressed with an increasing curve in the test range, represented by the equation Y = 612.66x^0.6004^, with a confidence level of 94%. Therefore, a strong correlation exists between the sectional porosity and average pore size.

Figure 7d shows the morphology of the internal pores according to the energy density. Small pores of hundreds of nanometres were created over the entire experimental range in the ASTM 316L specimens produced using the PBF process. 

Pores smaller than 12 μm were rare in specimens with an energy density of 155 J/mm^3^. Unmelted powder was not observed inside these pores, and its shape spherical. The size of the pores is smaller than the average particle size of the supplied powder. Hence, pores were generated by the partial trapping of gas during the deposition process.

The creation characteristics of pores for energy densities of 137 J/mm^3^ and 155 J/mm^3^ were similar. However, large pores with maximum lengths of 100 μm or longer were observed when the energy density was lower than 137 J/mm^3^. Unmelted powder particles were observed inside large pores. The lower the energy density, the larger the number of pores.

### 3.3. Surface Roughness Characteristics according to Energy Density

**Figure 8:**
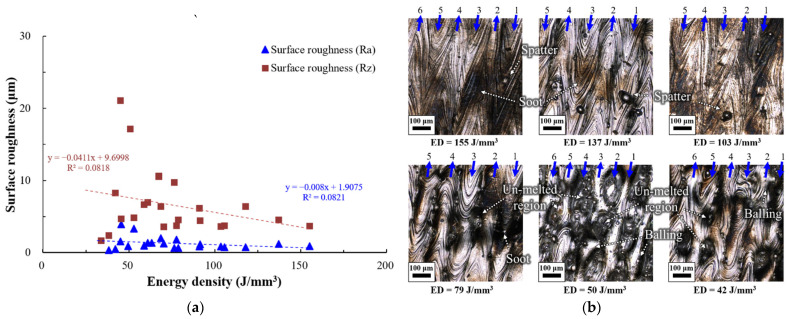
Surface roughness characteristics of deposited specimen for different energy density (J/mm^3^): (**a**) Plot of surface roughness (μm) according to energy density (J/mm^3^) and (**b**) Morphology of deposited surface according to the energy density (J/mm^3^).

The surface roughness properties of the 27 specimens produced using the PBF process were discussed in terms of the energy density used to fabricate the specimens. Figure 8a shows a scatter plot of the surface roughness according to the energy density of the specimens in terms of Ra and Rz. When the energy densities were 63 J/mm^3^, 49 J/mm^3^, and lower than or equal to 33 J/mm^3^, the surface roughness could not be measured because the specimen shape collapsed, or the surface state became unstable. The surface roughness, Ra, calculated with Equation (5), varied from 0.3 μm to 3.9 μm according to the energy density. Ra was lower than or equal to 1.2 μm for specimens with an energy density greater than or equal to 79 J/mm^3^. At the highest energy density of 155 J/mm^3^, Ra was 0.7 μm. Ra was unstable between 0.3 and 3.91 μm for an energy density lower than 79 J/mm^3^. Ra increased with increasing energy density, which can be expressed by the linear function Y = −0.008x + 1.9075, with a low confidence level of 8%.

The surface roughness, Rz, ranged from 1.6 μm to 21 μm according to the energy density. Rz was calculated with Equation (6) and varied between 1.7–21.1 μm according to the energy density condition. Rz was somewhat stable in the range of 3.65–6.38 μm when the energy density was 79 J/mm^3^ or higher, similar to Ra. Rz decreased with an increase in energy density. This behaviour can be expressed by the linear function Y = −0.0411x + 9.6998, with a low confidence level of 8%.

The low confidence level of function of Ra and Rz are calculated. This is because results with large dispersion (energy density level was 79 j/mm^3^ or less) were also considered. When the energy density level was 79 j/mm3 or less, irregular shapes with balling and collapse defects were observed. This defect could cause large dispersion of surface roughness data.

Figure 8b shows a confocal microscopy image of the ASTM 316L specimens according to the energy densities. The blue colour in the figure represents the order and direction of the melted beads. The deposition paths were readily observed on the surface shapes when the energy density was 79 J/mm^3^ or higher. However, they were difficult to observe for energy densities lower than 79 J/mm^3^ owing to their unstable shapes.

Flat surfaces without unmelted shapes or vacant regions between the beads were created when the energy density was 103 J/mm^3^ or higher. Surface soot or spatter is present under these conditions.

The soot was caused by a high heat input, becoming severe under high-energy-density conditions. No traces of direct laser emission were observed in the spatters. Therefore, the spatters on the deposition surface can be assumed to have been created from the beads created later and then delivered onto the already melted beads. These spatters were assumed to be the major cause of the pores observed when the energy density was 103 J/mm^3^ or higher, as shown in Figure 7.

When the energy density was 79 J/mm^3^, valleys were created intermittently between the beads. Partially melted powder particles were present in the valleys between beads. The bead shapes were difficult to verify. Numerous craters were created on the surface when the energy density was <50 J/mm^3^. The incompletely melted powder particles were irregularly sintered inside the craters. Some beads swelled like balls due to the balling phenomenon. These results indicate that the energy density must be 79 J/mm^3^ or higher to deposit ASTM 316L specimens with excellent surface roughness. The results also indicate that to create specimens with flat surfaces without an unmelted shape or vacant regions between beads, the energy density must be 103 J/mm^3^ or higher.

### 3.4. Hardness Characteristics according to Energy Density

**Figure 9:**
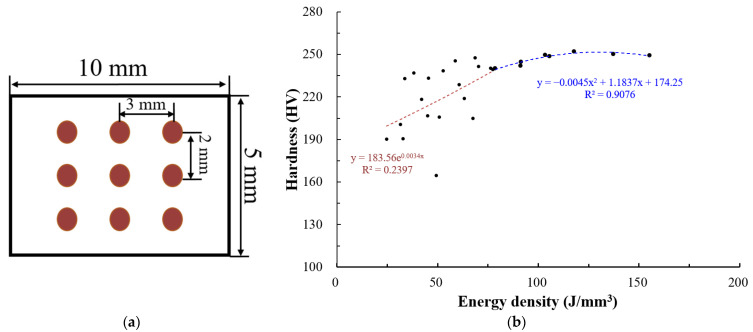
Hardness characteristics of deposited specimen for different energy density (J/mm^3^): (**a**) Measured location and (**b**) Plot of hardness (HV) according to energy density (J/mm^3^).

The hardness properties of ASTM 316L specimens produced by the PBF process are discussed. Figure 9a shows the hardness measurement sites. Figure 9b shows the hardness measurement results. The hardness properties of ASTM 316L specimens produced by the PBF process can be divided into two energy density ranges. The measured hardness in the energy density range of 79 J/mm^3^ or higher ranged from 240 HV to 252 HV. In this range, the higher the energy density, the higher the hardness. The increasing curve can be expressed by the quadratic function y = −0.0045x^2^ + 1.1837x +1 74.24, with a significant confidence level of 91%. When the energy density was lower than 79 J/mm^3^, the measured specimen hardness was 164~247 HV according to the energy density. In this range, the higher the energy density, the higher is the hardness. However, the confidence level is low (24%). The increasing curve can be expressed using the exponential function Y = 185.56 × 10 ^0.0034x^. The singularity of the hardness property is observed when the energy density is 79 J/mm^3^. The relative density is 99.5% or higher. The powder melts approximately completely when the energy density is 79 J/mm^3^ or higher, as shown in Figure 7 and Figure 8.

Considering that the hardness of ASTM 316L in ASTM standard, specimens perfectly constructed by the PBF process will have a hardness of approximately 20% higher than the standard level owing to rapid heating and cooling in the PBF process [17,19,22].

### 3.5. Tensile Characteristics According to Energy Density

**Figure 10:**
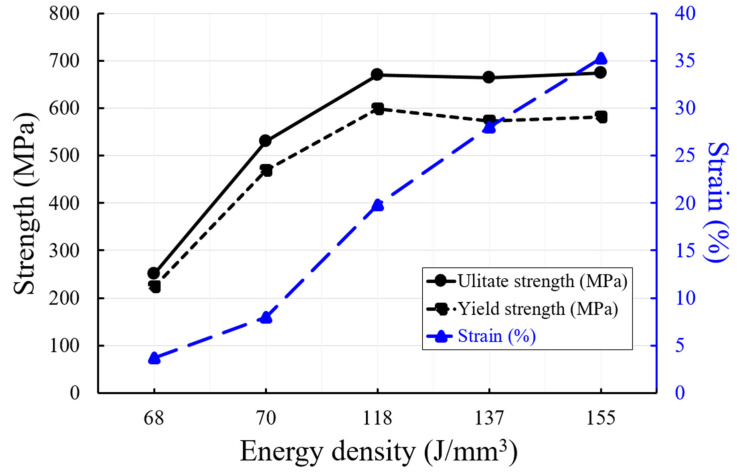
The ultimate strength, yield strength, and elongation variation curves according to the energy density (J/mm^3^).

Applying the energy density conditions used for examining the porosity, surface roughness, surface shape, and hardness properties, the ASTM 316L tensile specimens were produced by the PBF process. Their ultimate strength, yield strength, and elongation properties were discussed. The ultimate strength, yield strength, and elongation variation curves according to the energy density of ASTM 316L specimens produced by the PBF process are shown in Figure 10.

Singularity was observed at an energy density of 79 J/mm^3^ when discussing the results of porosity, surface roughness, surface shape, and hardness properties. Therefore, three and two conditions were selected in the energy density range higher than 79 J/mm^3^ and lower than 79 J/mm^3^, respectively, to produce tensile specimens for a total of five energy density conditions. Energy conditions of 50 J/mm^3^ or lower, where the specimens were not perfectly fabricated, were eliminated from the tensile specimen candidate list.

The three conditions selected in the energy density range above 79 J/mm^3^ were 118, 137, and 155 J/mm^3^. Under these three conditions, the yield strength and ultimate strength ranged from 573 to 582 MPa and 664–675 MPa, respectively. The average tensile and ultimate strengths in the energy density range greater than 79 J/mm^3^ were 585 MPa and 670 MPa, respectively. In this range, the tensile and ultimate strengths have rather stable properties, with negligible variations of less than 2% according to the energy density. The elongation distinctly increases with increasing energy density. If the energy density is increased by approximately 31% (from 118 J/mm^3^ to 155 J/mm^3^) in this range, the elongation improves 78% (from 19.8% to 35.3%). Therefore, the higher the energy density used in specimen deposition, the greater the improvement in elongation.

The two conditions selected in the energy density range below 79 J/mm^3^ were 68 and 70 J/mm^3^, respectively. The measured yield strength, ultimate strength, and elongation were 469 MPa, 530 MPa, and 8%, respectively, at an energy density of 70 J/mm^3^. The tensile specimens created with an energy density of 70 J/mm^3^ have reduced tensile and ultimate strengths by 21.4% and 19.3%, respectively, compared to those of the tensile specimens created with an energy density of 155 J/mm^3^. The elongation was also reduced by 77.3%.

The measured yield strength, ultimate strength, and elongation were 240 MPa, 251 MPa, and 3.7%, respectively, at an energy density of 68 J/mm^3^. The tensile specimens created with an energy density of 68 J/mm^3^ had reduced yield strength and ultimate strength by 62.8% and 61.5%, respectively, compared to those of the tensile specimens created with an energy density of 155 J/mm^3^. The elongation was also reduced by 89.5%.

These findings show that ASTM 316L tensile specimens can be fabricated under energy density conditions of 68 J/mm^3^ and 70 J/mm^3^ (lower than 79 J/mm^3^). However, their mechanical properties, such as yield strength, ultimate strength, and elongation, were insufficient. The average tensile and ultimate strengths were 585 MPa and 670 MPa, respectively, in the energy density range higher than or equal to 118 J/mm^3^. Whereas the elongation improved up to 78% as the energy density increased. The yield strength, ultimate strength, and elongation of ASTM 316L in ASTM standard were 206 MPa, 517 MPa, and 40%, respectively [19]. ASTM 316L specimens with tensile and ultimate strengths up to 183% and 29.6%, respectively, (higher than the requirement) can be produced without additional heating. In addition, specimens with 35.3% elongation (11.7% lower than the required elongation of 40%) can be produced.

### 3.6. Fracture Characteristics according to Energy Density

**Figure 11:**
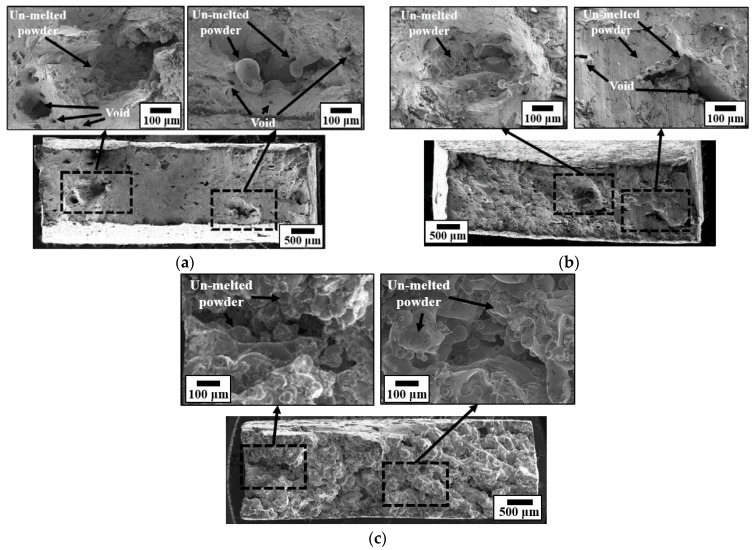
Images of the fracture surface for different energy densities (J/mm^3^): (**a**) energy density 155 J/ mm^3^; (**b**) energy density 118 J/mm^3^; and (**c**) energy density 68 J/mm^3^.

Images of the fracture surfaces were used to discuss the failure characteristics of the tensile specimens. Fracture characteristics of AISI 316L were presented in previous research work [21]. However, the effect of energy density on the fracture characteristics with examined energy density level was not discussed. Photographs of the cross-sections of the tensile specimens obtained using the FE-SEM equipment are shown in Figure 11. Figure 11a shows a picture of the fracture surface of the tensile specimen produced with an energy density of 155 J/mm^3^. Pores were observed on a part of the fracture surface. Unmelted powder particles were present inside the pores. The measured diameters of the particles ranged from 10 μm to 116 μm. Considering the size of the trapped particles, the 116 μm particles were likely spatter or balling. Other small particles were presumed to have not melted owing to uncertain variables such as powder supply imbalance during specimen production. Figure 11b shows a picture of the fracture surface of the tensile specimen produced with an energy density of 118 J/mm^3^. It is similar to that produced using 155 J/mm^3^. The low elongation under both conditions compared with the standard elongation of ASTM 316L was caused by the internal pores and powder particles that remained unmelted.

Figure 11c shows a picture of the fracture surface of the tensile specimen produced with an energy density of 68 J/mm^3^. The tensile specimens exhibited sintered bonding properties, without complete fusion. Powder particles sintered without complete fusion are observed on the fracture surfaces. In addition, numerous pores were observed in the cross-section. The low yield strength, ultimate strength, and elongation under these conditions were caused by the sintered bonding properties without complete fusion.

The measured pore size on the fracture surface of the tensile specimen was larger than that of the sectional porosity results. This was because the fracture occurred around the large pores inside the specimen during the tensile test process. The pores became coarse during the tensioning process.

**Figure 12:**
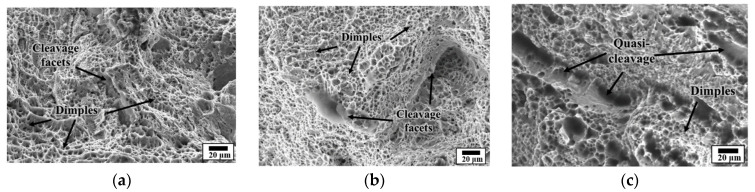
Fracture behaviour of deposited specimen for different energy density (J/mm^3^): (**a**) Energy density 155 J/ mm^3^; (**b**) Energy density 118 J/mm^3^; and (**c**) Energy density 68 J/mm^3^.

The results of analysing the fracture behaviours of the tensile specimens produced under the three energy density conditions are shown in Figure 12. When the energy density was 155 J/mm^3^, dimple structures caused by ductile fracture occupied most of the fracture surface, with some cleavage facets from brittle fractures. When the energy density was 118 J/mm^3^, dimple structures occupied most of the fracture surface. However, the cleavage facets caused by brittle fracture were coarse. When the energy density was 68 J/mm^3^, the cleavage ratio further increased to the level of dimple structures. Numerous quasi-cleavages were present. The variation in the fracture property according to the energy density was because the powder completely melted at a high energy density. However, the melting and bonding degrees between the powder particles were reduced at a low energy density. The yield and ultimate strengths were comparable when the energy densities were 155 J/mm^3^ and 118 J/mm^3^, respectively. However, the elongation varied significantly owing to the difference in the fracture properties. The low elongation of 3.7% with the sintered bonding property at an energy density of 68 J/mm^3^ was also caused by the unstable fracture property.

## 4. Conclusions

The objective of this study was to investigate the effects of energy density on the pore, hardness, surface roughness, and tensile characteristics of deposited ASTM 316L specimens with a powder bed fusion process. Twenty-seven types of cuboid specimens and five tensile specimens were fabricated. The characteristics of the pores, hardness, surface roughness, and tensile strength according to the energy density are discussed. The following conclusions were drawn from this study:

1. A significant correlation between the energy density used in specimen deposition and the sectional porosity of the specimens was observed, that is, the higher the energy density, the lower the porosity.
2. The energy density had to be at least 79 J/mm^3^ and 103 J/mm^3^, respectively, to produce ASTM 316L specimens with relative densities of 99.5% and 99.9%, respectively, using the PBF process.
3. A strong correlation was observed between the sectional porosity and average pore size.
4. The energy density had to be 79 J/mm^3^ or higher to deposit ASTM 316L specimens with excellent surface roughness. To create specimens with flat surfaces without an unmelted shape or vacant regions between beads, the energy density must be 103 J/mm^3^ or higher.
5. A singularity of the hardness property was observed when the energy density was 79 J/mm^3^ since the relative density was 99.5% or higher. Approximately complete melting takes place when the energy density is 79 J/ mm^3^ or higher.
6. ASTM 316L specimens with tensile and ultimate strengths up to 183% and 29.6%, respectively, higher than required, could be produced without additional heating. However, the elongation of deposited was 11.7% lower.
7. The yield and ultimate strengths were comparable when the energy densities were 155 J/ mm^3^ and 118 J/ mm^3^, respectively. The elongation varied significantly owing to the difference in the fracture properties. The low elongation of 3.7% with the sintered bonding property at an energy density of 68 J/ mm^3^ was also caused by the unstable fracture property.

This study used ASTM 316L material and PBF process with high industrial demand and also systematically investigated various mechanical properties according to a wide range of energy densities. Hence, it will be applicable to various industries. Future investigations on the effects of building direction and heat treatment on mechanical characteristics are needed to improve the mechanical properties of ASTM316L specimens.

## Figures and Tables

**Table 1:** Parameters and conditions for cuboid deposition experiments.

No	LP (W)	SS (mm/s)	W_B_ (mm)	H_D_ (mm)	OR (%)	ED (J/mm^3^)
1	200	500	172	86	50	155
2	150	500	146	73	50	137
3	100	500	113	57	50	118
4	200	1000	126	63	50	106
5	200	500	172	129	25	103
6	150	500	146	109	25	91
7	150	1000	110	55	50	91
8	100	500	113	85	25	79
9	200	500	172	172	0	78
10	200	1500	116	58	50	76
11	200	1000	126	95	25	70
12	150	500	146	146	0	69
13	150	1500	99	49	50	68
14	100	1000	105	53	50	63
15	150	1000	110	82	25	61
16	100	500	113	113	0	59
17	200	1000	126	126	0	53
18	200	1500	116	87	25	51
19	100	1500	90	45	50	49
20	150	1000	110	110	0	46
21	150	1500	99	74	25	45
22	100	1000	105	79	25	42
23	200	1500	116	116	0	38
24	150	1500	99	99	0	34
25	100	1500	90	67	25	33
26	100	1000	105	105	0	32
27	100	1500	90	90	0	25

**Table 2:** Chemical composition of deposition and substrate material (Wt.%).

Elements	Fe	Cr	Mo	Ni	C	P
ASTM 316L	Bal.	17.76	2.28	12.62	0.02	0.013
ASTM 1045	Bal.	0.028	0.005	0.005	0.47	0.026

## Data Availability

Not applicable.

## References

[1] Gor M., Soni H., Wankhede V., Sahlot P., Grzelak K., Szachgluchowicz I., Kluczyński J. (2021). A Critical Review on Effect of Process Parameters on Mechanical and Microstructural Properties of Powder-Bed Fusion Additive Manufacturing of SS316L. Materials.

[2] Ekoi E.J., Alessandrini G.D., Mughal M.Z., Vijayaraghavan R.K., Obeidi M.A., Groarke R., Kraev I., Krishnamurthy S., Brabazon D. (2022). Investigation of the Microstructure and Phase Evolution Across Multi Material Ni50.83Ti49.17-AISI 316L alloy interface fabricated using laser powder bed fusion (L-PBF). Mater. Des..

[3] Aota L.S., Bajaj P., Zilnyk K.D., Jägle E.A., Ponge D., Sandim H.R.Z., Raabe D. (2021). Recrystallization Kinetics, Mechanisms, and Topology in Alloys Processed by Laser Powder-Bed Fusion: AISI 316L Stainless Steel as Example. Materialia.

[4] Wang X., Muñiz-Lerma J.A., Attarian Shandiz M., Sanchez-Mata O., Brochu M. (2019). Crystallographic-Orientation-Dependent Tensile Behaviours of Stainless Steel 316L Fabricated by Laser Powder Bed Fusion. Mater. Sci. Eng. A..

[5] Afkhami S., Dabiri M., Piili H., Björk T. (2021). Effects of Manufacturing Parameters and Mechanical Post-Processing on Stainless Steel 316L Processed by Laser Powder Bed Fusion. Mater. Sci. Eng. A..

[6] Jiang H., Xi R., Li X., Kustov S., Humbeeck J.V., Wang X. (2022). Structure, Martensitic Transformation, and Damping Properties of Functionally Graded NiTi Shape Memory Alloys Fabricated by Laser Powder Bed Fusion. Metals.

[7] Calignano F., Minetola P. (2019). Influence of Process Parameters on the Porosity, Accuracy, Roughness, and Support Structures of Hastelloy X Produced by Laser Powder Bed Fusion. Metals.

[8] Greco S., Gutzeit K., Hotz H., Kirsch B., Aurich J.C. (2020). Selective Laser Melting (SLM) of AISI 316L—Impact of Laser Power, Layer Thickness, and Hatch Spacing on Roughness, Density, and Microhardness at Constant Input Energy Density. Int. J. Adv. Manuf. Technol..

[9] Herzog D., Bartsch K., Bossen B. (2020). Productivity Optimization of Laser Powder Bed Fusion by Hot Isostatic Pressing. Addit. Manuf..

[10] Fan H., Yang S. (2020). Effects of Direct Aging on Near-Alpha Ti–6Al–2Sn–4Zr–2Mo (Ti-6242) Titanium Alloy Fabricated by Selective Laser Melting (SLM). Mater. Sci. Eng. A..

[11] Aristizabal M., Jamshidi P., Saboori A., Cox S.C., Attallah M.M. (2020). Laser Powder Bed Fusion of a Zr-Alloy: Tensile Properties and Biocompatibility. Mater. Lett..

[12] Zhao J.R., Hung F.Y., Lui T.S. (2020). Microstructure and Tensile Fracture Behavior of Three-Stage Heat Treated Inconel 718 Alloy Produced via Laser Powder Bed Fusion Process. J. Mater. Res. Technol..

[13] Oliveira J.P., Lalonde A.D., Ma J. (2020). Processing Parameters in Laser Powder Bed Fusion Metal Additive Manufacturing. Mater Des..

[14] Ravichander B.B., Amerinatanzi A., Shayesteh Moghaddam N.S. (2020). Study on the Effect of Powder-Bed Fusion Process Parameters on the Quality of as-Built IN718 Parts Using Response Surface Methodology. Metals.

[15] Mumtaz K., Hopkinson N. (2009). Top Surface and Side Roughness of Inconel 625 Parts Processed Using Selective Laser Melting. Rapid Prototyp J..

[16] Cai C., Wu X., Liu W., Zhu W., Chen H., Qiu J.C.D., Sun C., Liu J., Wei Q., Shi Y. (2020). Selective Laser Melting of Near-α Titanium Alloy Ti-6Al-2Zr-1Mo-1V: Parameter Optimization, Heat Treatment and Mechanical Performance. J. Mater. Sci. Technol..

[17] Lo Y., Liu B., Tran H. (2019). Optimized Hatch Space Selection in Double-Scanning Track Selective Laser Melting Process. Int. J. Adv. Manuf. Technol..

[18] Murkute P., Pasebani S., Isgor O.B. (2019). Production of Corrosion-Resistant 316L Stainless Steel Clads on Carbon Steel Using Powder Bed Fusion-Selective Laser Melting. J. Mater. Process. Technol..

[19] ASTM. https://www.astm.org/a0240_a0240m-22.html.

[20] Peng T., Chen C. (2018). Influence of Energy Density on Energy Demand and Porosity of 316L Stainless Steel Fabricated by Selective Laser Melting. Int. J. Precis. Eng. Manuf. Green Technol..

[21] Liverani E., Toschi S., Ceschini L., Fortunato A. (2017). Effect of Selective Laser Melting (SLM) Process Parameters on Microstructure and Mechanical Properties of 316L Austenitic Stainless Steel. J. Mater. Process. Technol..

[22] Larimian T., Kannan M., Grzesiak D., Almangour B., Borkar T. (2020). Effect of Energy Density and Scanning Strategy on Densification, Microstructure and Mechanical Properties of 316L Stainless Steel Processed via Selective Laser Melting. Mater. Sci. Eng. A..

[23] Xu J., Ding Y., Gao Y., Wang H., Hu Y., Zhang D. (2021). Grain Refinement and Crack Inhibition of Hard-to-weld Inconel 738 Alloy by Altering the Scanning Strategy During Selective Laser Melting. Mater. Des..

